# Orthodoxies and Heresies

**Published:** 1916-04-01

**Authors:** 


### ORTHODOXIES AND HERESIES.

As there are no orthodoxies in medicine there cannot, of course, be any heresies. Doubtless there are doctrines which are widely received, and their general consent is obviously a fact of some moment. But every member of the profession is free to form an individual judgment on any question which attracts his attention, and this without risk of authoritative censure or penal consequence. Further, if he can give reasons for the faith that is in him, he may count on an audience and disciples. Granted that his aim is truth and his methods honest, he needs only perseverance to obtain victory. The way may be long if the proposals are revolutionary, but this experience is not peculiar to the world of medicine. What may fairly be claimed in medicine is that a challenge of ancient and generally received faiths is neither crushed by official thunder nor suppressed by the discipline of martyrdom. Such hindrance as exists meets reformers everywhere. Some will say it is a healthy conservatism; others will ascribe it to the hardness of men's hearts.

Of course freedom, here as elsewhere, has its disadvantages, and the line between use and abuse is not always easily defined. Certainly there is discomfort, more especially for those who love law and order, to know that at any moment their favourite faith or cherished opinion may be abruptly challenged. To find the convictions of a lifetime lightly thrown into the melting-pot is an experience which tests the patience of most men, and under it a scientific and impartial calm is not easily maintained. Yet there is no half-way house between the two positions. Either men must be free to think and speak the thing they will or they must be directed, and, if needs be, must be silenced, by authority. Medicine has had its experience of the latter discipline and has finally abandoned it as intolerable. Hence its adherents are no longer called upon either to swallow formulas or to shrink from new ideas. But escape from the shackles of authority and tradition at once opens the gateways of freedom, and with freedom comes danger to received opinions and settled creeds. To ask for the one without the other is a vain thing and belongs only to incompetence. In medicine, we take it, this is accepted as axiomatic, and most of us, however middle-aged we be, try to keep an open mind and what degree of patience we can command even to those who summon us to abandon our scientific convictions. All we ask, and all we ought to ask, is for good and substantial evidence from those who assail us. The onus, at least generally speaking, is on the innovators. "Use and wont" may not be scientific proof, but it certainly argues a capacity for existence, and this scores in its favour. There are nearly always startling new proposals in medicine, and no one would discourage the spirit which gives rise to them. That many of them come to no good end is true. But this is because of their own feebleness, not of the hostility of the environment into which they are born.

